# Structural and biochemical analysis of highly similar HLA-B allotypes differentially associated with type 1 diabetes

**DOI:** 10.1016/j.jbc.2024.107702

**Published:** 2024-08-22

**Authors:** Ruby Sharma, Nitin P. Amdare, Agnidipta Ghosh, Jennifer Schloss, John Sidney, Scott J. Garforth, Yessenia Lopez, Alev Celikgil, Alessandro Sette, Steven C. Almo, Teresa P. DiLorenzo

**Affiliations:** 1 Department of Microbiology and Immunology, Albert Einstein College of Medicine, Bronx, New York, USA; 2 Department of Biochemistry, Albert Einstein College of Medicine, Bronx, New York, USA; 3 Center for Infectious Disease and Vaccine Research, La Jolla Institute for Immunology, La Jolla, California, USA; 4 Division of Infectious Diseases and Global Public Health, Department of Medicine, University of California, La Jolla, California, USA; 5 Division of Endocrinology and Diabetes, Department of Medicine, Albert Einstein College of Medicine, Bronx, New York, USA; 6 Einstein-Mount Sinai Diabetes Research Center, Albert Einstein College of Medicine, Bronx, New York, USA; 7 Fleischer Institute for Diabetes and Metabolism, Albert Einstein College of Medicine, Bronx, New York, USA

**Keywords:** autoimmunity, crystallography, major histocompatibility complex (MHC), protein expression, type 1 diabetes

## Abstract

Type 1 diabetes (T1D) is an autoimmune disease involving T cell-mediated destruction of the insulin-producing beta cells in the pancreatic islets of Langerhans. CD8^+^ T cells, responding to beta cell peptides presented by class I major histocompatibility complex (MHC) molecules, are important effectors leading to beta cell elimination. Human leukocyte antigen (HLA) B∗39:06, B∗39:01, and B∗38:01 are closely related class I MHC allotypes that nonetheless show differential association with T1D. HLA-B∗39:06 is the most predisposing of all HLA class I molecules and is associated with early age at disease onset. B∗39:01 is also associated with susceptibility to T1D, but to a lesser extent, though differing from B∗39:06 by only two amino acids. HLA-B∗38:01, in contrast, is associated with protection from the disease. Upon identifying a peptide that binds to both HLA-B∗39:06 and B∗39:01, we determined the respective X-ray structures of the two allotypes presenting this peptide to 1.7 Å resolution. The peptide residues available for T cell receptor contact and those serving as anchors were identified. Analysis of the F pocket of HLA-B∗39:06 and B∗39:01 provided an explanation for the distinct peptide C terminus preferences of the two allotypes. Structure-based modeling of the protective HLA-B∗38:01 suggested a potential reason for its peptide preferences and its reduced propensity to present 8-mer peptides compared to B∗39:06. Notably, the three allotypes showed differential binding to peptides derived from beta cell autoantigens. Taken together, our findings should facilitate identification of disease-relevant candidate T cell epitopes and structure-guided therapeutics to interfere with peptide binding.

Type 1 diabetes (T1D) is an organ-specific autoimmune disease involving the demise of the insulin-producing beta cells in the pancreatic islets of Langerhans, leading to insulin insufficiency. In this T cell-mediated disease, autoreactive CD8^+^ and CD4^+^ T cells respond to beta cell peptides that are presented in the context of class I or class II major histocompatibility complex (MHC) molecules, respectively (1). Multiple peptides derived from beta cell autoantigens, such as insulin and glucose-6-phosphatase 2 (G6Pase 2), have been identified as T cell epitopes in T1D (2). The disease is a polygenic condition; however, the human leukocyte antigen (HLA) genomic region, encoding the MHC molecules of humans, shows the strongest association (3), consistent with the T cell-mediated nature of the disease. Structural studies of MHC molecules associated with susceptibility or resistance to T1D have been pivotal for elucidating the underlying mechanisms. For example, X-ray crystallography of MHC class II molecules predisposing to T1D in humans (4) or the nonobese diabetic (NOD) mouse model of the disease (5, 6) revealed their unique peptide-binding characteristics. This structural knowledge continues to yield benefits, including assisting in the development of structure-guided T1D therapies (7, 8). However, parallel studies of MHC class I molecules associated with T1D have lagged behind. Indeed, it has been nearly two decades since HLA-B∗39:06 was initially shown to be predisposing to T1D and associated with younger age at onset (9), but the structure of the allotype encoded by this MHC allele has not yet been reported. This is a critical area of study, given the mounting evidence that cytotoxic CD8^+^ T cells are important effectors in beta cell elimination (10, 11, 12, 13, 14, 15), coupled with the observation that T1D is associated with hyperexpression of class I MHC on beta cells (16).

HLA class I molecules consist of an allele-specific heavy chain that is associated noncovalently with β2-microglobulin (β2m) and a presented peptide. The α1 and α2 domains of the heavy chain form the peptide-binding groove, which is characterized by six pockets designated A-F. In general, two well-conserved primary anchor residues occupy the B and F pockets and are located at position two and the C terminus of the peptide, respectively (17). HLA class I alleles can be grouped into supertypes based on similarities in their peptide-binding grooves and shared primary anchor residues (18, 19). Members of a supertype are predicted to show overlap in their peptide-binding repertoires (20). HLA-B∗39:06, B∗39:01, and B∗38:01 are highly similar (greater than 97% amino acid identity) and are all members of the B27 supertype (19), yet they are differentially associated with T1D. Remarkably, HLA-B∗39:06 is the most predisposing of all HLA class I alleles, while B∗38:01 is the most protective (21, 22). Like HLA-B∗39:06, B∗39:01 is also associated with susceptibility to T1D, yet its effect is less pronounced (9, 22, 23, 24, 25). We have used a combination of X-ray crystallography, modeling, and peptide-binding analyses to study these three HLA-B allotypes and investigate the characteristics that underlie their differential association with T1D. The findings further enhance our understanding of disease pathogenesis and have implications for the development of targeted therapeutic strategies.

## Results

### Identification of an endogenous ligand of HLA-B∗39:06 that also binds HLA-B∗39:01

**Figure 1:**
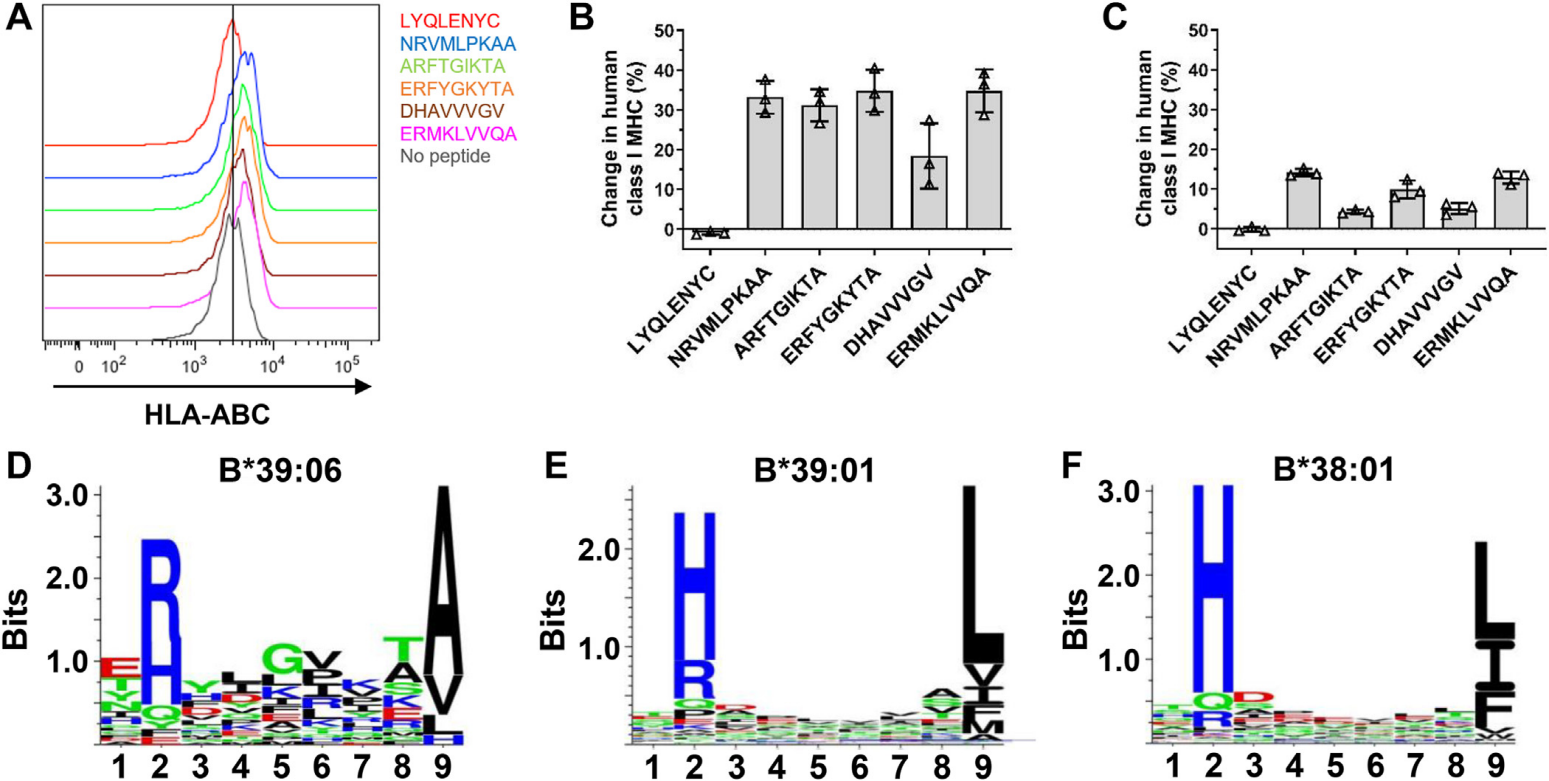
**Peptide-binding characteristics of closely related HLA-B allotypes.***A-C*, C1R/HLA-B∗39:06 cells (*A* and *B*) or C1R/HLA-B∗39:01 cells (*C*) were incubated with the indicated peptides at 26 °C overnight and changes in the level of human class I MHC relative to no peptide added were assessed by flow cytometry. *A*, representative flow cytometry plot. *Vertical line* indicates mean fluorescence intensity = 3000. *B* and *C*, mean change in level of human class I MHC; *triangles* represent technical replicates; error bars represent SD. *D-F*, peptide-binding motifs for HLA-B∗39:06 (*D*), B∗39:01 (*E*), and B∗38:01 (*F*). Peptide ligands for each HLA-B allotype were compiled using the Immune Epitope Database (72), with the majority of the ligands being peptides eluted from MHC molecules purified using the antibody W6/32 (73), which is specific for a monomorphic HLA class I determinant. Sequence logos for 9-mer peptides were constructed using Seq2Logo (74). At each position of the peptide, the more frequent residues are toward the *top*. A large letter indicates a frequent (*i.e.*, preferred) amino acid. Basic residues are shown in *blue*, acidic in *red*, hydrophobic in *black*, and hydrophilic in *green*. HLA, human leukocyte antigen; MHC, major histocompatibility complex.

**Figure 2:**
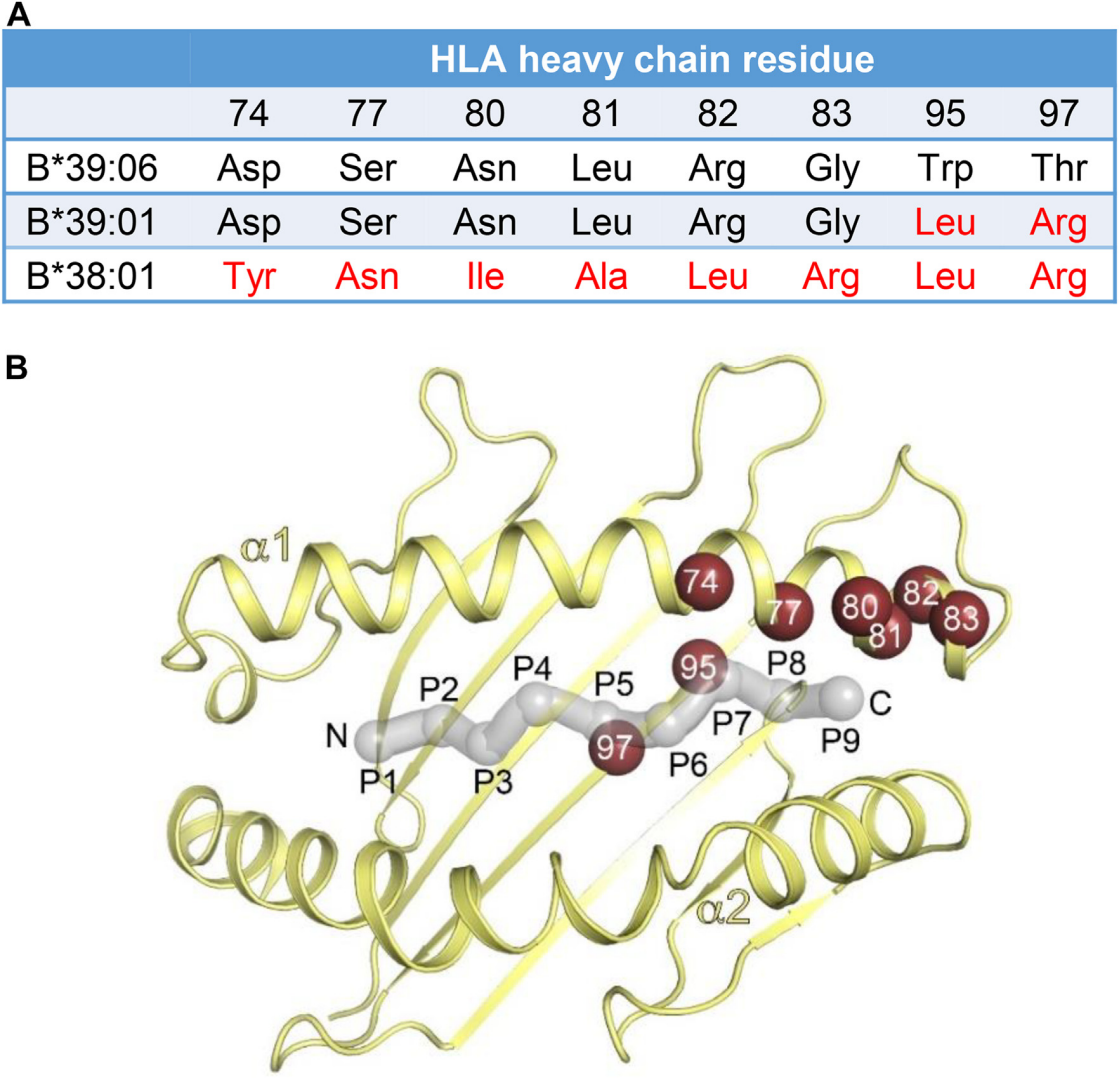
**Amino acid sequence comparison of HLA-B∗39:06, B∗39:01, and B∗38:01.***A*, the three allotypes are identical at all positions except those shown. Differences between HLA-B∗39:06 and B∗39:01 or B∗38:01 are shown in *red*. *B*, a view of the peptide-binding groove of HLA-B∗39:06 (*yellow*) as reported here and shown as a *ribbon* with *arrows* for β strands and *ribbons* for helices (PDB 9C6V). The α1 and α2 helices are labeled. The peptide NRVMLPKAA is depicted as a *gray* transparent tube and *spheres* (Cα), with peptide positions labeled P1-P9. Cα positions of MHC residues that differ between HLA-B∗39:06 and B∗39:01 or B∗38:01 are numbered and shown as *red spheres*. HLA, human leukocyte antigen; MHC, major histocompatibility complex.

We previously reported the sequences of peptides endogenously bound to and eluted from HLA-B∗39:06 molecules purified from the human lymphoblastoid cell line C1R/HLA-B∗39:06 (26). We developed a peptide-binding assay utilizing C1R/HLA-B∗39:06 cells to validate the binding of several of these. Our strategy was based on the prior seminal observations of Ploegh et al. (27) that the incubation of cells at 26 °C allows for empty class I MHC molecules to reach the cell surface, and that the MHC can be stabilized if a binding peptide is also included. C1R/HLA-B∗39:06 cells were incubated with each candidate peptide at 26 °C overnight, and the change in the amount of human class I MHC relative to no added peptide was assessed by flow cytometry (Fig. 1, *A* and *B*). The binding of each candidate HLA-B∗39:06 binding peptide was confirmed based on the enhancement of human class I MHC expression. Importantly, the irrelevant insulin-derived peptide LYQLENYC resulted in no such increase. As additional validation for the assay, a cell-free competitive peptide-binding assay using purified HLA-B∗39:06 and a high-affinity radiolabeled ligand identified DHAVVVGV as by far the weakest HLA-B∗39:06 binder among the candidates ((26) and data not shown), as also observed in the cell-based assay (Fig. 1, *A* and *B*). These same endogenous ligands of HLA-B∗39:06 were also tested for their ability to bind to B∗39:01, which differs from B∗39:06 only at residues 95 and 97 (Fig. 2, *A* and *B*), using C1R/HLA-B∗39:01 cells in our cell-based assay (Fig. 1*C*). All test peptides were able to bind to some degree to HLA-B∗39:01; however, the overall magnitude of binding was less compared to HLA-B∗39:06. This difference is consistent with our observation that the peptide-binding motifs for the two HLA-B allotypes are not identical (Fig. 1, *D* and *E*). Both prefer Arg or His at peptide position two; however, at the C-terminal position of the peptide, HLA-B∗39:06 prefers Ala while B∗39:01 prefers Leu.

For elucidation of the crystal structures of HLA-B∗39:06 and B∗39:01, the peptide selected was NRVMLPKAA, which represents amino acids 320 to 328 of human NACHT, LRR, and PYD domains-containing protein 2 (NLRP2). This peptide was chosen because it bound to both HLA-B∗39:06 and B∗39:01 in the cell-based assay (Fig. 1, *A*-*C*). These results were confirmed with a cell-free competitive peptide-binding assay using purified HLA-B∗39:06 and B∗39:01 and a high-affinity radiolabeled ligand. In this assay, NLRP2 320 to 328 bound to HLA-B∗39:06 with an IC_50_ of 14 nM and to B∗39:01 with an IC_50_ of 35 nM (Table 1). We reasoned that this peptide choice would potentially allow us to obtain and compare the structures of the two similar MHC molecules with an identical peptide.

**Table 1:** MHC binding capacity of the peptides relevant to the structural determinations or comparisons in this report

Peptide source	Peptide	Position	Length	IC_50_ (nM)^a^		
				B∗39:06	B∗39:01	B∗38:01
Human NLRP2	NRVMLPKAA	320	9	**14**	**35**	-b
	NRVMLPKA	320	8	**41**	**85**	*n*.*d*.
Human DDX3X	SHVAVENAL	2	9	696	**11**	**321**

Peptide binding was quantitatively measured based on the inhibition of binding of a high-affinity radiolabeled peptide to purified MHC molecules as described in Experimental procedures.

*n*.*d*., not determined.

a IC_50_ values ≤ 500 nM are shown in bold (strong to intermediate binding).

b A dash indicates IC_50_ > 50,000 nM (no binding).

### Crystal structure of HLA-B∗39:06

**Figure 3:**
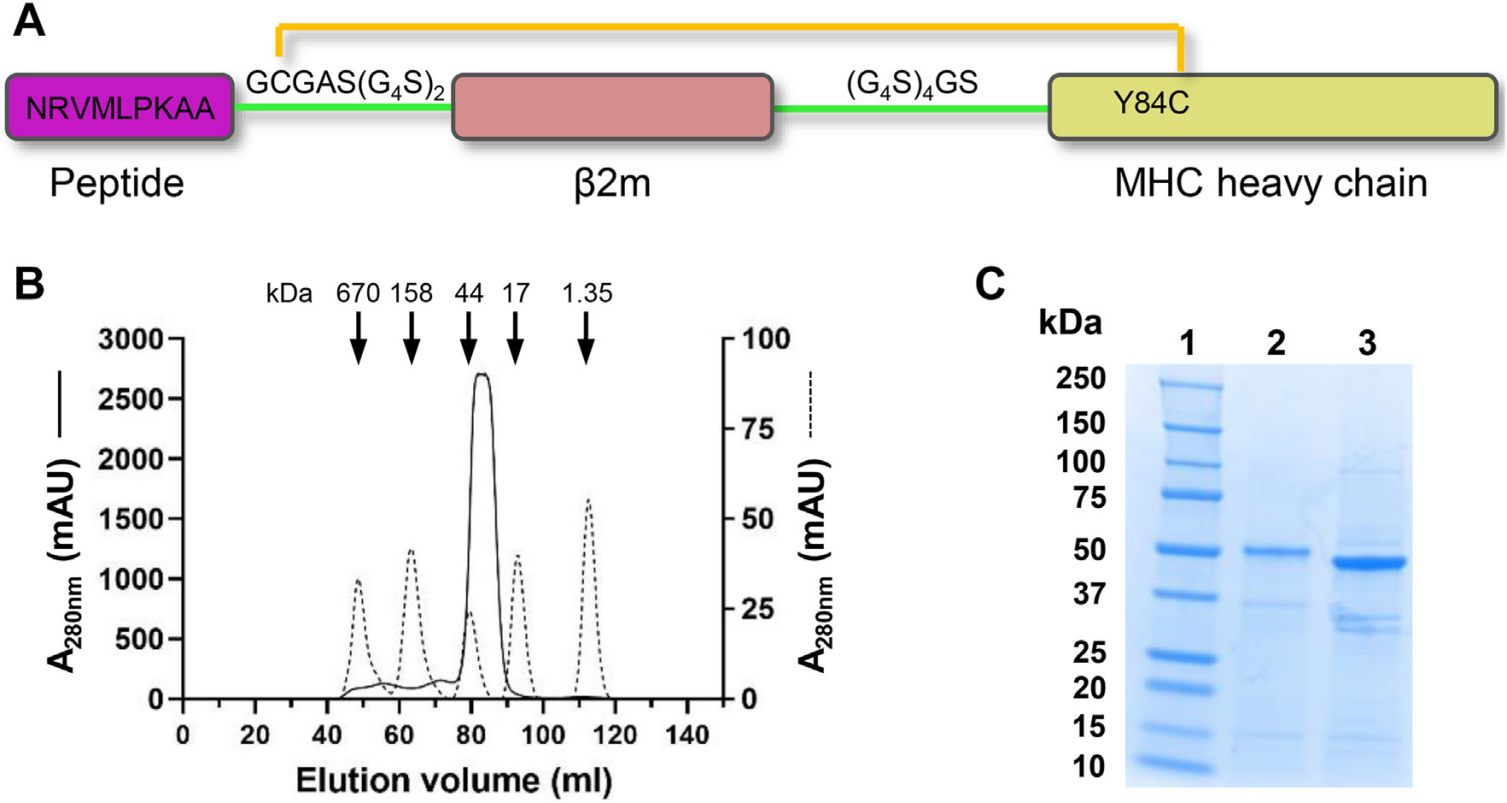
**Expression and purification of HLA-B∗39:06 as a single-chain secreted protein.***A*, schematic representation of the expression construct (not to scale). The peptide (NRVMLPKAA) is shown in *purple*, β2m in *red*, and the ectodomain of the HLA-B∗39:06 heavy chain in *yellow*. Shown in *green* are linkers between the C terminus of the peptide and the N terminus of β2m and between the C terminus of β2m and the N terminus of the HLA-B∗39:06 heavy chain. The engineered disulfide between the Cys of the first linker and Y84C of the heavy chain is depicted in *gold*. *B*, stable FreeStyle 293-F cell lines were established to secrete HLA-B∗39:06/NRV into the culture media. The supernatant was passed through a Ni^2+^-NTA column followed by size-exclusion chromatography as described in Experimental procedures, and the chromatogram shown as a *solid blackline* was obtained. The *dotted black line* shows the chromatogram for protein standards having the indicated molecular weights. *C*, protein purity was assessed by SDS-PAGE under reducing conditions and Coomassie blue staining. *Lane 1*, protein molecular weight marker; *lane 2*, purified HLA-B∗39:06/NRV; *lane 3*, purified HLA-B∗39:06/NRV treated with PNGase F. β2m, β2-microglobulin; HLA, human leukocyte antigen.

**Figure 4:**
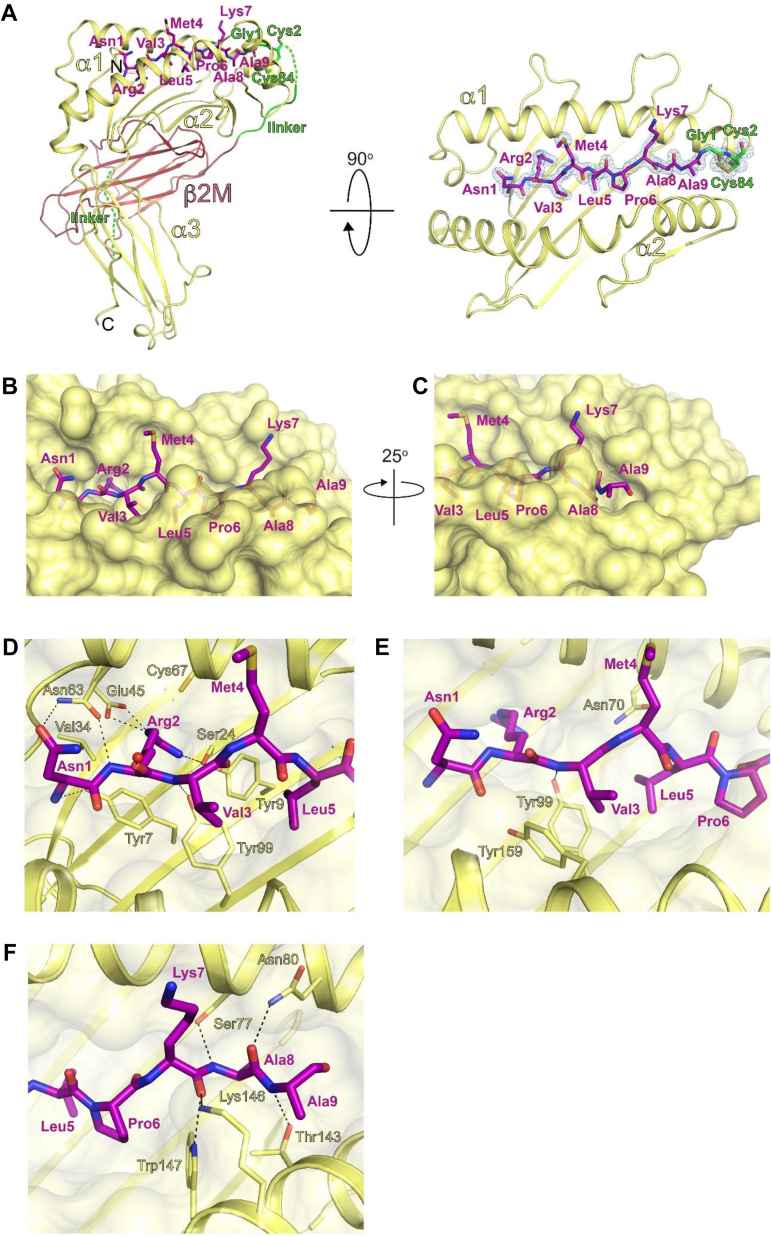
**Crystal structure of HLA-B∗39:06/NRV.***A*, *left*, a view of HLA-B∗39:06/NRV showing the HLA-B∗39:06 heavy chain (*yellow*), β2m (*red*), and the NRV peptide (*purple* and labeled; stick representation). Shown in *green* are linkers between the C terminus of the peptide and the N terminus of β2m and between the C terminus of β2m and the N terminus of the heavy chain. The engineered disulfide between the Cys of the first linker and Y84C of the heavy chain is depicted in *gold*. The α1, α2, and α3 domains of the MHC heavy chain are labeled. N and C denote the location of N and C termini. *Right*, an orthogonal view of the peptide-binding groove of HLA B∗39:06 along with the bound NRV peptide. Omit map corresponding to the NRV peptide is shown in *blue mesh* (σ = 2.5). *B*, close-up view of the NRV peptide (stick representation; *purple*) bound to HLA-B∗39:06 (transparent surface representation; *yellow*) highlights that the side chains of Arg2, Val3, Leu5, Pro6, and Ala8 are buried in the peptide-binding groove, and the side chains of Asn1, Met4, and Lys7 are solvent-exposed. *C*, rotated close-up view shows that the side chain of Ala9 of the NRV peptide is solvent-exposed. *D*, a view of interactions between Arg2, the primary anchor residue present at peptide position 2 (P2), and the MHC. Interacting side chains of HLA-B∗39:06 are shown in stick representations (*yellow*) and labeled. The *red sphere* denotes a water molecule that interacts with the Arg2 main chain. *E*, Interactions between Val3 (a secondary anchor residue) of the bound peptide with HLA-B∗39:06. Side chains of the MHC involved in either polar or hydrophobic interactions are shown in *stick* representations and labeled. *F*, a view of the interactions between Ala8 of the peptide with HLA-B∗39:06. Atomic contacts are indicated by *dashed lines*. β2m, β2-microglobulin; HLA, human leukocyte antigen; MHC, major histocompatibility complex.

**Figure 5:**
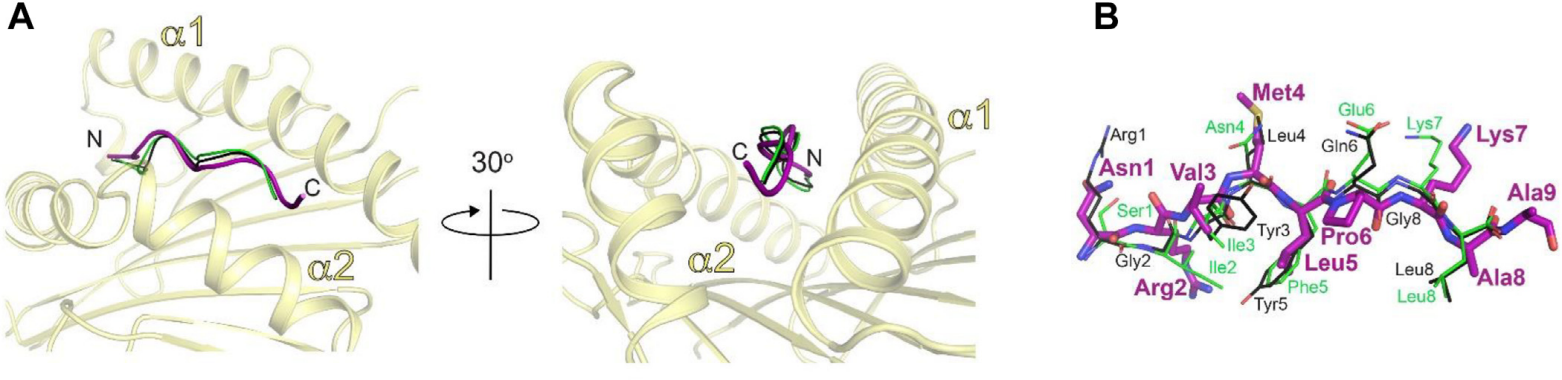
**The NRV peptide binds to HLA-B∗39:06 as an 8mer.***A*, comparison (cartoon representations) of the 8-mer peptides OVA-8 (SIINFEKL, *green*, PDB 1VAC) and VSV-8 (L4V) (RGYLYQGL, *black*, PDB 1OSZ), as bound to H2-K^b^, with the NRV peptide (*purple*) bound to HLA-B∗39:06 (*yellow*). The comparison was made by overlaying the heavy chains of H2-K^b^ (not the bound peptides) onto HLA-B39∗06. *B*, stick representations of the NRV, OVA-8, and VSV-8 peptides (colored as in *A*), with side chain positions labeled. HLA, human leukocyte antigen; PDB, Protein Data Bank.

Many available crystal structures of class I MHC molecules have been obtained using soluble class I MHC heavy chain and β2m refolded from bacterial inclusion bodies in the presence of free peptide. However, this strategy was unsuccessful in our hands for HLA-B∗39:06. Thus, to obtain the protein in quantities suitable for X-ray crystallography, HLA-B∗39:06 was produced in mammalian cells as a secreted single-chain peptide-MHC (sc-pMHC) in which the NRVMLPKAA peptide, human β2m, and the extracellular domain of the HLA-B∗39:06 heavy chain were joined with flexible linkers (Fig. 3*A*). To enhance protein stability, a substitution was made at position 84 of the MHC heavy chain (Y84C) (28, 29, 30), which leads to the formation of a disulfide bond between Cys84 and an additional cysteine introduced at the second position of the linker connecting the peptide and β2m (Fig. 3*A*). This HLA-B∗39:06 complex was expressed in FreeStyle 293-F cells and purified from the culture supernatant using nickel affinity and size-exclusion chromatography (Fig. 3*B*). Class I MHC heavy chains have an N-linked glycosylation site at Asn86 (31). Though not expected to interfere with crystallization (32), the glycan was nonetheless removed with PNGase F (Fig. 3*C*). Using this material, we determined the crystal structure of HLA-B∗39:06 to 1.7 Å resolution (Protein Data Bank (PDB) 9C6V; Figs. 4*A*, S1, and Table S1). Electron density for the engineered disulfide bond was clearly visible (Fig. S2*A*). Consistent with the finding that Arg at position two of the peptide is a preferred residue for HLA-B∗39:06 (Fig. 1*D*), the structure revealed that the side chain of this anchor residue is located in the B pocket (Fig. 4*B*) and makes polar contacts with B-pocket residues Ser24 and Glu45 of the B∗39:06 heavy chain (Figs. 4*D*, S3, and Table S2). The F pocket of a class I MHC molecule normally engages the C-terminal anchor residue of the peptide. However, in our structure, Ala8 of NRVMLPKAA, and not Ala9, occupies the F pocket and forms polar and van der Waals contacts with F-pocket residues Ser77, Asn80, Trp95, Thr143, and Trp147 (Figs. 4*F*, S3, and Table S2), while Ala9 is solvent-exposed (Fig. 4*C*). Although nine amino acids is the predominant length of the endogenous ligands of HLA-B∗39:06, approximately 20% are 8mers (26, 33), including the known insulin-derived T cell epitope MRLLPLLA (34, 35). Importantly, the 8-mer peptide NRVMLPKA bound well to both HLA-B∗39:06 and B∗39:01 in our cell-free competitive binding assay, exhibiting an IC_50_ of 41 nM and 85 nM, respectively (Table 1). Based on this behavior, it is plausible to postulate a model in which covalently tethered NRVMLPKAA bound analogously to an 8mer, with Ala at position nine functioning as the *de facto* first residue of the linker between the bound 8mer and β2m. To probe this hypothesis, we compared the placement of NRVMLPKAA within B∗39:06 with that of the *bona fide* 8-mer peptides OVA-8 (SIINFEKL; PDB 1VAC; (36)) and VSV-8 (L4V) (RGYLYQGL; PDB 1OSZ; (37)) when noncovalently bound to H2-K^b^. The peptide-binding grooves of the heavy chains of H2-K^b^ and HLA-B∗39:06 were superimposed, which resulted in RMSDs between 0.54 Å (over 170 aligned Cα atoms of PDB 1OSZ) and 0.74 Å (over 169 aligned Cα atoms of PDB 1VAC), and the positioning of the peptides was compared (Fig. 5, *A* and *B*). This superposition was possible due to the structural conservation between H2-K^b^ and HLA class I MHC molecules (17). Superposition of H2-K^b^ and HLA-B∗39:06 underscores the 8-mer peptides OVA-8 and VSV-8 overlay with the first eight amino acids of the NRVMLPKAA peptide with RMSDs of 0.95 Å and 0.84 Å, respectively, over the eight aligned Cα atoms. This analysis suggests that the covalently tethered 9-mer peptide NRVMLPKAA binds to HLA-B∗39:06 analogous to an 8mer, with Ala8 occupying the F pocket. Additionally, our HLA-B∗39:06 structure suggests that for 8-mer peptides presented by this HLA-B allotype, the residues that are solvent-exposed and likely available to participate in interactions with a cognate T cell receptor (TCR) are at positions four and seven (Met4 and Lys7; Fig. 4, *B* and *C*). Previous characterization of the peptide-binding specificity of HLA-B∗39:06 using single amino acid-substituted peptides suggested that peptide position three functions as a secondary anchor (26). Consistent with this behavior, we found that Val3 of the peptide forms van der Waals contacts with multiple residues of the MHC heavy chain (Fig. S3 and Table S2), in addition to a hydrogen bond with Tyr99 (Fig. 4*E*). Importantly, the above structural features of HLA-B∗39:06 were also observed in a second independent crystal form (Fig. S1 and Table S1; PDB 9C6W).

### Structural comparison of HLA-B∗39:06 and B∗39:01

HLA-B∗39:01 differs from B∗39:06 only at residues 95 and 97 (Fig. 2, *A* and *B*), yet the two allotypes are differentially associated with T1D, with B∗39:06 conferring the greater risk (9, 22, 23, 24, 25). To understand this difference at a structural level, we sought to compare the two highly similar MHC molecules presenting an identical peptide. To this end, HLA-B∗39:01 was produced as a sc-pMHC (Fig. S4*A*), including the covalently linked NRVMLPKAA peptide, as described above for HLA-B∗39:06. Using this material, we determined the crystal structure of HLA-B∗39:01 to 1.7 Å resolution (PDB 9C6X; Figs. S2*B*, S4, *B* and *C*, S5, Tables S1, and S3). The peptide was positioned as in the HLA-B∗39:06 structure, with the first eight amino acids (NRVMLPKA) occupying the peptide-binding groove (Fig. S4). For both allotypes, Arg2 and Ala8 serve as the primary anchor residues, and Met4 and Lys7 are available for contact with a TCR.

**Figure 6:**
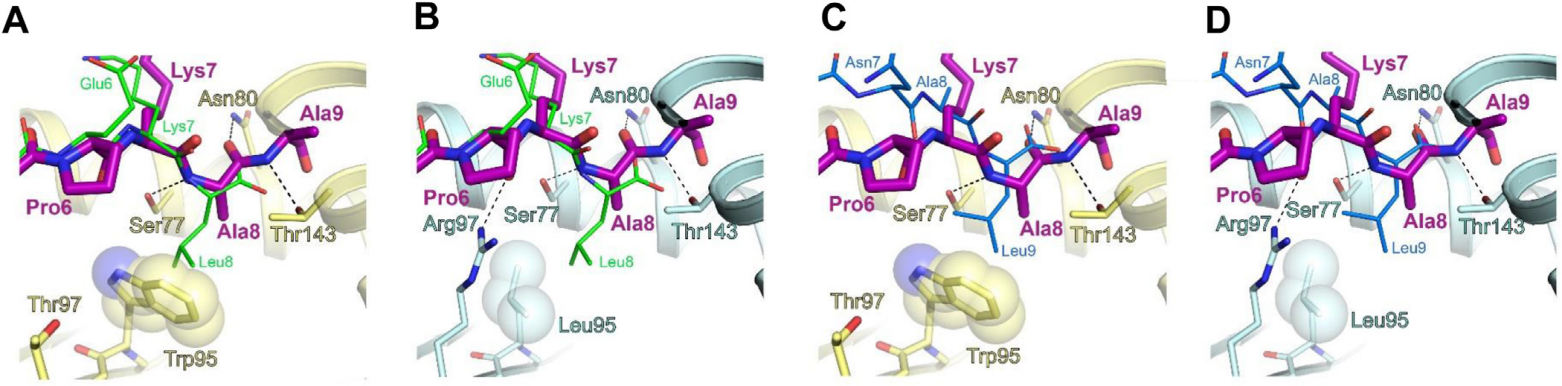
**HLA-B∗39:06 prefers shorter hydrophobic side chains in the F pocket.***A*, close-up view of the interaction of the NRV peptide (stick representation, *purple*) with the F pocket of HLA-B∗39:06 (*yellow*). Interacting side chains of the MHC are shown in stick representation and labeled. The Trp95 side chain is shown as sticks with transparent spheres. The OVA-8 peptide (PDB 1VAC) is overlaid (stick representation, *green*) to illustrate that Leu8 is not preferred by the Trp95 side chain of B∗39:06. *B*, in contrast, Leu95 (stick representation with transparent spheres) in the F pocket of HLA-B∗39:01 (*cyan*) more easily accommodates larger hydrophobic side chains at the C terminus of a peptide (*e.g.*, Leu8 of OVA-8). *C*, close-up view of the F pocket of HLA-B∗39:06 highlights the side chain of Leu9 of the DDX3X peptide (SHVAVENAL, *blue*, PDB 4O2E) is less favored in the pocket due to the presence of Trp95. *D*, a view of the HLA-B∗39:01 F pocket highlighting that Leu95 readily accommodates C-terminal hydrophobic side chains that are larger than Ala, as shown for Leu9 of the DDX3X peptide. DDX3X, DEAD-box helicase 3 X-linked; HLA, human leukocyte antigen; MHC, major histocompatibility complex; PDB, Protein Data Bank.

The F pocket of a class I MHC molecule generally accommodates the C-terminal residue of the presented peptide (17). As F-pocket residue 95 differs between HLA-B∗39:06 (Trp) and B∗39:01 (Leu), we concentrated our structural comparison of the two allotypes on this region. Overlaying the OVA-8 peptide SIINFEKL (PDB 1VAC; (36)) on our HLA-B∗39:06 structure illustrates that the indole side chain of Trp95 provides limited space in the F pocket for Leu at peptide position eight (Fig. 6*A*). This observation explains, at least in part, why the smaller Ala is preferred at the C terminus of peptides presented by HLA-B∗39:06 (Fig. 1*D*). In contrast, Leu95 in HLA-B∗39:01 allows the F pocket to more easily accommodate hydrophobic side chains larger than Ala (Fig. 6*B*), consistent with the preference for Leu at the C terminus of the bound peptide (Fig. 1*E*). To further explore this point, we took advantage of a previously reported structure of HLA-B∗39:01 that was produced by refolding of bacterial inclusion bodies of heavy chain and β2m with the 9-mer peptide SHVAVENAL derived from DEAD-box helicase 3 X-linked (DDX3X) (38). Superimposing the HLA-B∗39:01/SHVAVENAL coordinates (PDB 4O2E) on our HLA-B∗39:06 and B∗39:01 structures once again highlights that the side chain of Leu at the C-terminus of the peptide is less favored in the F pocket of HLA-B∗39:06 due to the presence of the bulky Trp95 (Fig. 6, *C* and *D*) and is more readily accommodated by HLA-B∗39:01 (Fig. 6*D*). The other dimorphic residue between HLA-B∗39:06 and B∗39:01, though not in the F pocket, is at position 97. In HLA-B∗39:01, Arg97 can hydrogen-bond to the carbonyl group of Pro6 of the NRV peptide (Fig. 6*B*), while Thr97 of B∗39:06 is unable to do so (Fig. 6*A*).

### Comparative modeling analysis of HLA-B∗39:06 and B∗38:01

**Figure 7:**
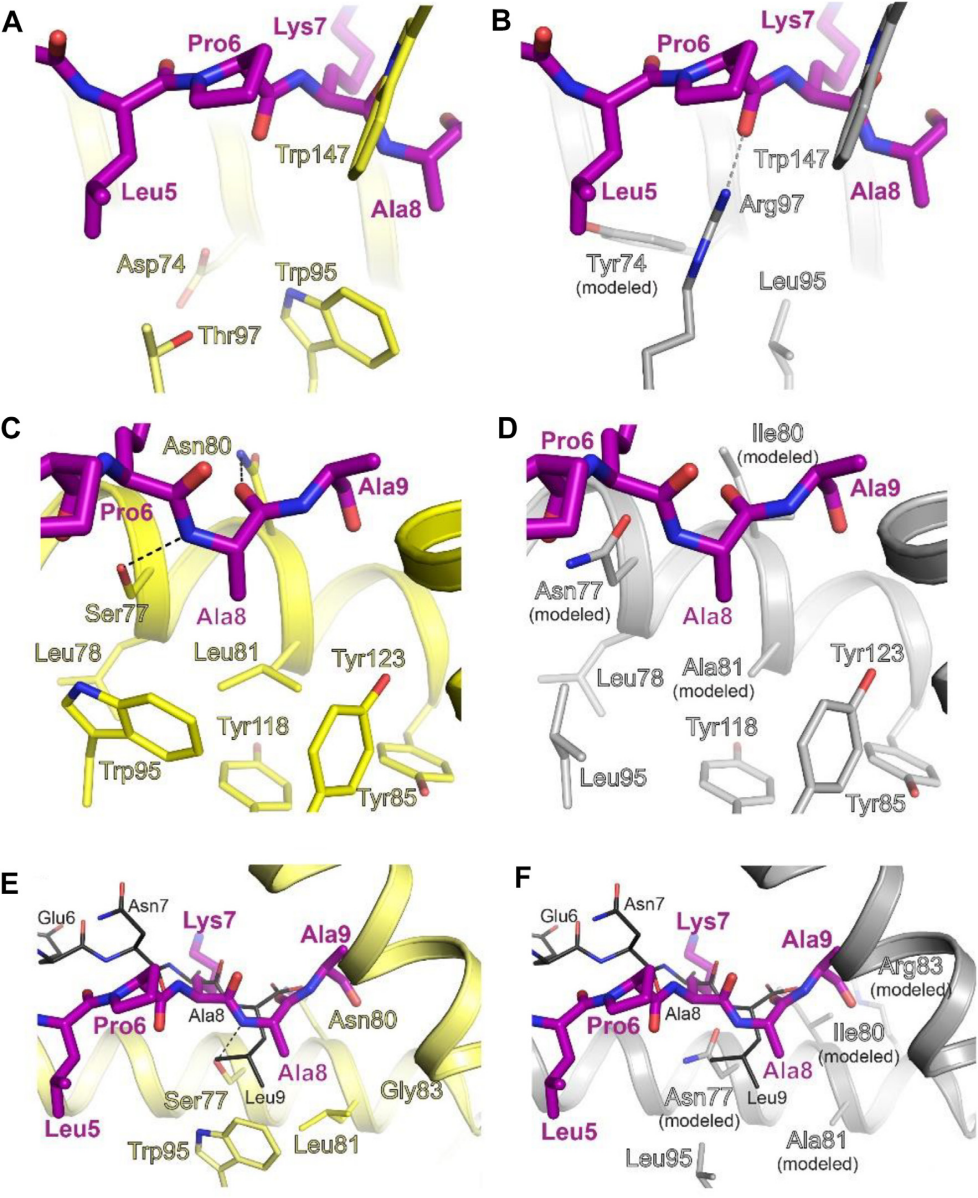
**Differences in HLA-B allotypes and their preferences in the peptide-binding groove.***Left* panels, HLA-B∗39:06 (PDB 9C6V, *yellow*); *right* panels, HLA-B∗38:01 (modeled, *gray*). The NRV peptide is shown as a stick representation (*purple*) in all panels. *A* and *B*, close-up view suggests Tyr74 (modeled in stick representation, *gray*) in B∗38:01 may disfavor a large hydrophobic side chain at position five of an 8-mer peptide (*B*), while Asp74 in B∗39:06 (*A*) allows it. *C* and *D*, view of the F pocket suggests Asn77 (modeled, *gray*) in B∗38:01 (*D*) will not allow an 8-mer peptide to bind in the conformation that is observed in *C* for B∗39:06. Additionally, in B∗38:01, Ile80 and Ala81 (both modeled) make the F pocket of the MHC potentially better suited for accepting a large hydrophobic sidechain at the C terminus of the peptide (*i.e.*, where Ala8 of the NRV peptide is positioned in the HLA-B∗39:06 structure). *E* and *F*, extended view of the F pocket, with the 9-mer peptide SHVAVENAL (PDB 4O2E) included as a stick representation (*black*). In HLA-B∗39:06 (*E*), Leu is less favored in the pocket due to the presence of Trp95. In contrast, Leu95 in B∗38:01 readily accommodates hydrophobic side chains at the peptide C-terminus that are larger than Ala, as shown for Leu9 of SHVAVENAL. HLA, human leukocyte antigen; MHC, major histocompatibility complex; PDB, Protein Data Bank.

Differential peptide binding of the closely related HLA-B allotypes B∗39:06 and B∗39:01 was suggested by the peptide sequence logos depicted in Fig. 1, *D* and *E*, and explained, at least in part, by our structural studies (Fig. 6). In the case of HLA-B∗39:06 and B∗38:01, differences in binding preferences were suggested by peptide sequence logos (Fig. 1, *D* and *F*; and (33)), and supported by binding studies using single amino acid-substituted peptide libraries (26). To explore the structural basis for the differential peptide binding of HLA-B∗39:06 and B∗38:01, HLA-B∗38:01 allotype-specific amino acid side chains were modeled in COOT (39, 40) (https://www.ccp4.ac.uk/) using refined coordinates of HLA-B∗39:06/NRV as a template (Fig. 7). This strategy was used because the structure of HLA-B∗38:01 has not been reported. For this analysis, it is important to keep in mind that in our HLA-B∗39:06/NRV structure, Ala9 of the NRV peptide is behaving as the first residue of the linker between the peptide and β2m; thus, the peptide binds as an 8mer with Ala8 occupying the F pocket. Our modeling suggests that Tyr74 in B∗38:01 may disfavor a large hydrophobic side chain at position five of an 8-mer peptide (Fig. 7*B*), while Asp74 in B∗39:06 (Fig. 7*A*) allows it. Furthermore, study of the F pocket suggests Asn77 in HLA-B∗38:01 (Fig. 7*D*) will not allow an 8-mer peptide to bind in the conformation observed for B∗39:06 (Fig. 7*C*). These observations help to explain the finding that HLA-B∗39:06 has a greater propensity to bind 8mers than does B∗38:01 (33). Also, in B∗38:01, Ile80, Ala81, and Leu95 make the F pocket of the MHC potentially better suited for accepting a large hydrophobic sidechain at the C terminus of the peptide (Fig. 7*D*). We then examined the F pocket in the context of the 9-mer peptide SHVAVENAL (PDB 4O2E; (38)). Leu95 in B∗38:01 readily accommodates hydrophobic side chains at the peptide C-terminus that are larger than Ala, as shown for Leu9 of SHVAVENAL (Fig. 7*F*). However, in HLA-B∗39:06 (Fig. 7*E*), Leu is less favored in the pocket due to the presence of Trp95. Importantly, we acknowledge that future work will be needed to validate our modeling-based assertions.

### Binding of autoantigen-derived peptides to HLA-B∗39:06, B∗39:01, and B∗38:01

HLA-B∗39:06 and B∗38:01 are closely related and differ by only eight amino acids (Fig. 2, *A* and *B*). Yet, HLA-B∗39:06 is the HLA class I allele that is the most strongly associated with T1D susceptibility, while B∗38:01 is the most protective (21, 22). We reasoned that the differential ability of the HLA-B allotypes to mediate T1D might be related to their differential binding and presentation of disease-relevant beta cell peptides. Given the apparent essential nature of T cell responses to insulin for the development of T1D (41), as well as the importance of T cells specific for G6Pase 2 in the preclinical NOD mouse model (42, 43, 44), we investigated the binding of peptides derived from these autoantigens to the closely related HLA-B allotypes (B∗39:06, B∗39:01, and B∗38:01). The majority of the peptides selected for study were identified as potential HLA-B∗39:06 binders, as detailed in Experimental procedures. Of the 95 human peptides examined for binding to the three HLA-B allotypes, eleven bound to at least one allotype with an IC_50_ ≤ 500 nM (strong to intermediate binding; Table 2). Of the seven peptides that satisfied this criterion for HLA-B∗39:06, all failed to meet the affinity criterion for B∗38:01. These results demonstrate that the susceptibility and protective allotypes show differential binding to peptides derived from important beta cell autoantigens.

**Table 2:** Human insulin and G6Pase 2 peptides binding to HLA-B∗39:06, B∗39:01, or B∗38:01

Peptide source	Peptide	Position	Length	IC_50_ (nM)^a^		
				B∗39:06	B∗39:01	B∗38:01
Ins	WMRLLPLLA	4	9	**155**	865	-b
	MRLLPLLA	5	8	**41**	**62**	44464
	MRLLPLLAL	5	9	677	**14**	850
	SHLVEALYL	33	9	35594	5253	**10**
G6Pase 2	NHSSPCLEQF	92	10	-	25661	**66**
	THFPHQVIL	173	9	2602	**73**	**142**
	TNLFLFLFAV	210	10	**354**	3949	6689
	FLFLFAVG	213	8	**405**	17882	-
	FRLLCALTSL	292	10	**7.4**	**290**	2756
	YHFLQIPT	307	8	**151**	660	9748
	SASIPLTVVA	328	10	**310**	30923	-

Peptide binding was quantitatively measured based on the inhibition of binding of a high-affinity radiolabeled peptide to purified MHC molecules as described in Experimental procedures.

a IC_50_ values ≤ 500 nM are shown in bold (strong to intermediate binding).

b A dash indicates IC_50_ > 50,000 nM (no binding).

## Discussion

Crystallographic analysis of secreted sc-pMHC produced in mammalian cells allowed us to compare the structures of HLA-B∗39:06 and B∗39:01 when presenting the same peptide. The single-chain construction was adapted from prior work, which demonstrated that an engineered disulfide between Y84C of the MHC heavy chain and a Cys in the linker between the peptide and β2m prevented peptide exchange and improved stability (28, 29, 30). Despite the known ability of bacterially produced disulfide-trapped sc-pMHC to be refolded and analyzed by X-ray crystallography (28), structural analysis of such proteins produced in mammalian cells has been limited (45). Our work reinforces the utility of this approach.

The peptide engineered into the sc-pMHC constructs studied here was NRVMLPKAA, a natural ligand eluted from HLA-B∗39:06 (26). We anticipated that Ala at position nine of the peptide would occupy the F pocket, which normally engages the C-terminal residue of the bound peptide. Unexpectedly, Ala at position eight of the peptide occupied the F pocket, with the Ala at position nine appearing to serve as part of the linker between the peptide and β2m. Subsequently, using our cell-free competition assay, we found that the 8mer NRVMLPKA bound well to both HLA-B∗39:06 and B∗39:01 (Table 1), consistent with our structural findings.

Because the 8-mer (NRVMLPKA) and 9-mer (NRVMLPKAA) peptides exhibited similar binding affinities in the competition assay, we sought an explanation for why only the first eight amino acids of the peptide (*i.e.*, NRVMLPKA) contacted the binding groove in the crystal forms we examined. Previously, HLA-B∗39:01 was crystallized with the 9mer SHVAVENAL using bacterially produced heavy chain and β2m and free peptide (38). In that case, the peptide exhibited a central bulge, required to place the C-terminal Leu in the F pocket and establish the conserved hydrogen bonding network that stabilizes class I MHC-bound peptides (46). One of the hydrogen bonds in this network is between the Tyr84 side chain and the terminal carboxyl group of the peptide. This interaction is disrupted in the sc-pMHC configuration due to the Y84C mutation and the lack of a carboxyl group on position nine of the peptide. Thus, for the sc-pMHC, creating a peptide bulge to place position nine in the F pocket may provide limited benefit and may even be less energetically favored and/or less amenable to crystal formation than having only the first eight amino acids occupying the groove. An alternative explanation for why the peptide bound only as an apparent 8mer in our structures could be that the engineered disulfide prohibited the peptide from binding as a 9mer. However, the existing literature on disulfide-trapped sc-pMHC complexes does not lend support to this idea, as the strategy has been used with 9-mer peptides and multiple MHC molecules from mice and humans (29, 30, 45, 47) and, importantly, correct 9mer presentation by these complexes has been verified using T cell recognition (29, 30, 47) and structural analysis (45).

It remains possible that NRVMLPKAA also binds unconventionally to HLA-B∗39:06 in nature, *i.e.*, with the penultimate Ala in the F pocket. Though the instance of peptide C termini extending out of the peptide-binding groove is rare, examples do exist (48, 49, 50). Interestingly, the sequence logo for 9-mer ligands of HLA-B∗39:06 shows a preference for Ala not only at position 9, but also at position eight (Fig. 1*D* and (33)), as did binding studies using single amino acid-substituted peptide libraries (26). These observations suggest that at least some 9-mer peptides bind B∗39:06 in the manner observed in our structure. Further investigation of this notion is warranted. The isolation of peptide-specific TCRs from HLA-B∗39:06-positive donors and the use of structurally defined reagents for antigen-specific T cell modulation and detection could help to clarify this point. Despite this uncertainty, our structural studies nonetheless provide important information regarding how an 8mer peptide, such as NRVMLPKA, may bind to the T1D-susceptibility allotypes HLA-B∗39:06 and B∗39:01.

Our structural and biochemical investigations of HLA-B∗39:06, B∗39:01, and B∗38:01 provide mechanistic insight into the differential association of these allotypes with T1D. Taken together, our results support the hypothesis that sequence and/or structural properties of HLA-B∗39:06 and, to a lesser extent, HLA-B∗39:01, are uniquely configured to present critical beta cell peptides to T cells to promote disease. One such peptide could be the 8mer MRLLPLLA (derived from the insulin leader sequence), which is recognized by peripheral blood T cells in T1D patients expressing B∗39:06 (35). Notably, the protective HLA-B∗38:01 binds few 8mers (33), and our modeling provides a likely structural explanation for this.

Regarding our results concerning the ability of peptides derived from beta cell autoantigens to bind to the HLA-B allotypes studied here (Table 2), one must appreciate that peptide binding is a double-edged sword in terms of T1D pathogenesis. On the one hand, a beta cell-derived peptide must exhibit some level of binding to MHC in order to activate CD8^+^ T cells that kill beta cells. However, if the peptide is from an antigen like insulin that is expressed in the thymus (51, 52), strong-binding peptides may induce T cell negative selection in the thymus if the peptide is presented there. As a result, even if the peptide were also presented by beta cells, T cells specific for such a peptide would likely not be present in the periphery and, thus, could not contribute to beta cell elimination. Future work must explore which of the identified HLA-B∗39:06-binding peptides are presented by human islets, and whether they are recognized by islet-infiltrating T cells isolated from T1D donors. Our HLA-B∗39:06-transgenic NOD mice (53), including lines engineered to express human beta cell autoantigens such as insulin (54), will also be useful for the identification of disease-relevant B∗39:06-restricted T cell specificities.

In conclusion, knowledge of the structure of the T1D susceptibility alleles HLA-B∗39:06 and B∗39:01 should yield practical benefits, including the ability to perform structure-guided identification of candidate T cell epitopes or strategies to interfere with peptide binding. Inspiration comes from recent advances in the structure-guided development of reagents that block peptide binding to class II MHC molecules that predispose to T1D, including H2-A^g7^ in mice (7) and HLA-DQ8 in humans (7). Besides T1D, HLA-B∗39:06 is also predisposing for Graves’ disease (55) and celiac disease (56), while B∗39:01 is associated with susceptibility to psoriatic arthritis (57). Thus, our findings could enhance our understanding of disease pathogenesis and have implications for the development of targeted therapeutic strategies for multiple autoimmune conditions.

## Experimental procedures

### Cell-based assessment of HLA-B∗39:06 and B∗39:01 peptide binding

The C1R cell line (58, 59), validated using short tandem repeat profiling, was obtained from American Type Culture Collection (CRL-1993). C1R cells engineered to express HLA-B∗39:06 (26) or HLA-B∗39:01 were generated by electroporation and selection in 2 mg/ml Geneticin. Several previously reported peptide binding assays (60, 61) were modified for use with HLA-B∗39:06 and HLA-B∗39:01. C1R/HLA-B∗39:06 or C1R/HLA-B∗39:01 cells were washed twice with PBS and then suspended in ice-cold PBS and cooled on ice for 5 min. Following centrifugation, cells were suspended in 1 ml ice-cold PBS and kept on ice for 1.5 min. Cells were washed with 49 ml serum-free Iscove's modified Dulbecco's medium (IMDM), pelleted, and washed once with serum-free IMDM. Cells were suspended in serum-free IMDM in the presence of 1.25 μM human β2m and 10 μM peptide of interest. Cells were incubated overnight at 26 ˚C and monitored for changes in the level of HLA-B∗39:06 or B∗39:01 by flow cytometry using an anti-HLA-ABC FITC-conjugated mAb (B9.12.1) (Beckman Coulter).

### Cell-free competitive peptide-binding assay

HLA-B molecules were isolated from a single MHC allele-transfected C1R cell line or an Epstein-Barr virus-transformed homozygous cell line, or were refolded from bacterial inclusion bodies as described (26, 62). Assays to quantitatively measure peptide binding to purified B∗39:06 and B∗39:01 molecules were based on the inhibition of binding of a high-affinity radiolabeled peptide and performed as described (26). Briefly, 0.1 to 1 nM of radiolabeled FRYQGHVGA (*Homo sapiens* proteasome subunit 127–135; IC_50_ 24 nM for B∗39:06), an endogenously bound and eluted ligand for B∗39:06 (26), was coincubated at room temperature with approximately 10 nM of purified MHC in the presence of a cocktail of protease inhibitors, 1 μM human β2m, and a test peptide. Following a 2-day incubation, MHC-bound radioactivity was determined by capturing MHC/peptide complexes on W6/32 (anti-class I) antibody coated Lumitrac 600 plates (Greiner Bio-One) and measuring bound cpm using the TopCount (Packard Instrument Co) microscintillation counter. In the case of competitive assays, the concentration of the test peptide yielding 50% inhibition of the binding of the radiolabeled peptide was calculated. Under the conditions utilized, where [label] < [MHC] and IC_50_ ≥ [MHC], the measured IC_50_ values are reasonable approximations of the true K_d_ values. Each competitor peptide was tested at six different concentrations covering a 100,000-fold dose range, and in three or more independent experiments. As a positive control, the unlabeled version of the radiolabeled probe was also tested in each experiment. Peptide binding to HLA-B∗38:01 was measured similarly, except that YHIPGDTLF (Variola virus RNA helicase 346–354; IC_50_ 1.4 nM) was used as the radiolabelled probe (26).

Candidate peptides of 8 to 10 residues derived from human preproinsulin and G6Pase 2 were tested for binding to HLA-B∗39:06, B∗39:01, and B∗38:01. The peptides were identified as potential HLA-B∗39:06 binders based on conformation with the primary anchor motif defined from sequencing endogenously bound ligands (26, 33). Additional peptides were selected using multiple bioinformatics-based prediction approaches. These included NetMHCpan (63); a matrix based on single amino acid substitution analysis (26); a positional scanning combinatorial library-based matrix (Table S4) derived and implemented as described (64); and a consensus approach using all three methods. Additional nonpredicted peptides were also included. In total, 95 peptides were selected (Table S5), and synthesized as crude material on a 1 mg scale by TC Peptide Lab.

### Mammalian expression constructs for sc-pMHC

Protein coding sequences were cloned into a modified version of pIRES-acGFP (Clontech) preceded by a human erythropoietin signal peptide. The HLA-B∗39:06 and B∗39:01 constructs encode a soluble sc-pMHC. The NLRP2 320 to 328 peptide (NRVMLPKAA) and human β2m are joined with a linker having the sequence GCGAS(G_4_S)_2_. A substitution was made at position 84 of the MHC heavy chain (Y84C) (29), which leads to the formation of a disulfide bond between the introduced Cys, and the Cys at position two of the linker between the peptide and β2m. β2m and the MHC heavy chain are linked by a (G_4_S)_4_GS linker, and the constructs contain a tobacco etch virus protease-cleavable 10xHis affinity tag.

### Expression and purification of soluble sc-pMHC

The expression constructs encoding sc-pMHC for HLA-B∗39:06 or B∗39:01 were transfected into FreeStyle 293-F cells (Thermo Fisher Scientific) using polyethylenimine (Polysciences) at a ratio to DNA of 4:1 by weight. Selection with 800 μg/ml G418 (InvivoGen) was started at 24 h posttransfection. G418 concentration was reduced to 100 μg/ml when cell viability decreased to ∼20 to 30%. Selected cells were expanded in 5-L flasks (Thomson). At a cell density of ∼3 × 10^6^/ml, one-third volume of fresh media was added, and valproic acid (Sigma-Aldrich) was added to a final concentration of 3 mM. Cell viability was monitored daily, and cells were harvested by centrifugation 7 days post transfection, or when viability was reduced to < 70%.

Proteins were purified from the culture supernatant by metal-chelate chromatography. His60 Ni Superflow Resin (Takara) was added to the supernatant to give a bed volume of 10 ml/L, and incubated with shaking for 1 h at room temperature. The resin was collected in a glass column by gravity flow, washed with >10 bed volumes of 25 mM Mes, 150 mM NaCl, 10% glycerol, 50 mM Arg-Cl, 5 mM imidazole, pH 6.5, and eluted with five bed volumes of 25 mM Mes, 150 mM NaCl, 10% glycerol, 100 mM Arg-Cl, 500 mM imidazole, pH 6.5. Proteins were concentrated using Amicon centrifugal units (EMD Millipore). The concentrated HLA-B∗39:06 and B∗39:01 proteins were purified using size-exclusion chromatography (Superdex 16/60 200 pg column; GE HealthCare) equilibrated with HBS-E buffer (20 mM Hepes, 150 mM NaCl, and 1 mM EDTA, pH 7.0). Further, HLA-B∗39:06 and B∗39:01 proteins were deglycosylated with PNGase F (New England BioLabs) at 37 °C for 12 to 14 h. The deglycosylated proteins were purified by subtractive chromatography using ConA beads (Sigma-Aldrich) and underwent a further round of size-exclusion chromatography equilibrated with HBS-E buffer. Fractions containing protein were pooled and concentrated using Amicon centrifugal units. Proteins were analyzed by SDS-PAGE (Bio-Rad) and gel images captured using a ChemiDoc Touch Imaging System (Bio-Rad). Protein concentrations were determined using UV absorbance at 280 nm; extinction coefficient was calculated from amino acid sequence using ProtParam (Expasy).

### Crystallization, structure determination, and refinement

Initial screening of the complexes (286 μM) was performed using 800 nl (protein:mother liquor = 1:1) sitting drops with a Crystal Gryphon (Art Robbins Instruments) utilizing MCSG-1 sparse matrix crystallization suite (Molecular Dimensions). Diffraction-quality crystals of HLA-B∗39:06/NRV were obtained in sitting drops by vapor diffusion against well solutions containing either 0.2 M potassium sulfate and 20% PEG 3350 (space group C222_1_; Table S1) or 2 M ammonium sulfate, 0.2 M lithium sulfate, and 0.1 M Hepes (pH 7.5) (space group P2_1_2_1_2_1_). Crystals were cryopreserved with 20% ethylene glycol prior to flash-cooling in liquid nitrogen. Diffraction-quality crystals of HLA-B∗39:01/NRV were obtained (sitting drops) against well solutions containing 25% PEG 3350 and 0.1 M Tris (pH 8.5). Crystals were cryopreserved with 10% ethylene glycol.

Data were collected with a CCD Pixel Dectris Pilatus 6M detector (wavelength 0.98 Å) on the ID-31 (LRL-CAT) beamline at the Argonne National Laboratory or with a CCD Pixel Dectris Eiger X 9M detector (wavelength 0.92 Å) on the NSLS-II beamline 17-ID-1 (AMX) at the Brookhaven National Laboratory (Table S1). Data from single crystals were integrated and scaled using AIMLESS (39). Diffraction of HLA-B∗39:06/NRV crystals was consistent with either of the orthorhombic space groups C222_1_ (a = 74.9, b = 83.5, c = 148.6 Å; one molecule/asymmetric unit; chain A) or P2_1_2_1_2_1_ (a = 75.3, b = 84.2, c = 149.2 Å; two molecules/asymmetric unit; chain A & B) and extended to resolutions of 1.68 Å and 1.67 Å, respectively. The HLA-B∗39:01/NRV complex was crystallized in space group C222_1_ (a = 75.9, b = 84.1, c = 150.4 Å; one molecule/asymmetric unit; chain A) and diffracted to 1.7 Å. Initial phases of the complexes were determined by molecular replacement with PHASER (65) (https://www.ccp4.ac.uk) using refined coordinates of HLA-B∗39:01/SHVAVENAL (PDB 4O2E) (38) as the search model. After determining the phases, atomic models were built into the density using the automated model building program BUCCANEER (66) (https://www.ccp4.ac.uk) and manually inspected using COOT (39, 40). The model was refined with REFMAC5 (39, 67) (https://www.ccp4.ac.uk) and PHENIX (68) (https://phenix-online.org). Analyses of the structures were performed in COOT and MOLPROBITY (69) (https://phenix-online.org). The crystallographic model exhibited excellent geometry with no residues in disallowed regions of the Ramachandran plot (70). LigPlot+, Version 2.2 (71) (http://www.ebi.ac.uk/thornton-srv/software/LigPlus) was used to generate two-dimensional contact maps between the NRV peptide and the HLA-B allotypes. Crystallographic statistics and Research Collaboratory for Structural Bioinformatics accession codes are provided in Table S1.

### Structure-based modeling of HLA-B∗38:01

HLA-B∗38:01 allotype-specific amino acid side chains were modeled using refined coordinates of HLA-B∗39:06/NRV as a template in COOT, followed by rigid body and real space refinement. Structure figures were generated using the PyMOL Molecular Graphics System, Version 2.5 (Schrödinger, LLC; https://www.pymol.org).

## Data availability

The structures presented in this article have been submitted to the Protein Data Bank under accession numbers 9C6V, 9C6W, and 9C6X. The resources and other data generated and analyzed during the current study are available from the corresponding authors upon reasonable request.

## Supporting information

This article contains supporting information.

## Conflict of interest

The authors declare that they have no conflicts of interest with the contents of this article.
