# Supplementary material for: Structural and biochemical analysis of highly similar HLA-B allotypes differentially associated with type 1 diabetes
Source: J Biol Chem. 2024 Aug 22;300(9):107702. doi: 10.1016/j.jbc.2024.107702 (PMC11422593; doi:10.1016/j.jbc.2024.107702)
Supplement: Supporting Figures S1-S5 and Tables S1-S5 [file mmc1.pdf]

Structural and biochemical analysis of highly similar HLA-B allotypes differentially associated with type 1 diabetes

Ruby Sharma, Nitin P. Amdare, Agnidipta Ghosh, Jennifer Schloss,

John Sidney, Scott J. Garforth, Yessenia Lopez, Alev Celikgil, Alessandro Sette,

Steven C. Almo, and Teresa P. DiLorenzo

Supporting Information

Material included:
Tables S1-S5
Figures S1-S5

Table S1. Crystallization conditions and crystallographic data statistics

HLA	B*39:06 (1 mol/asu)	B*39:06 (2 mol/asu)	B*39:01
**Peptide**	NRVMLPKAA	NRVMLPKAA	NRVMLPKAA
**Crystallization condition**	20% PEG 3350, 0.2 M Potassium sulfate	2M Ammonuim sulfate,0.2 M Lithium sulfate, 0.1 M HEPES pH 7.5	25% PEG 3350, 0.1 M Tris pH 8.5
**Cryo preservation**	20% (v/v) Ethylene glycol	20% (v/v) Ethylene glycol	10% (v/v) Ethylene glycol
**Data Collection***			
Source	APS 31-ID	APS 31-ID	BNL 17-ID-1
Wavelength (Å)	0.98	0.98	0.92
Number of crystals	1	1	1
Space group	$C222_1$	$P2_12_12_1$	$C222_1$
Cell dimensions			
a,b,c (Å)	74.95, 83.45, 148.63	75.24, 84.23, 149.2	75.94, 84.12, 150.42
Resolution (Å)	24.77 - 1.68 (1.7 - 1.68)	19.73 - 1.67 (1.69 - 1.67)	24.83 - 1.7 (1.73 - 1.7)
Completeness (%)	95.3 (92.9)	99.8 (99.8)	99.9 (99.9)
Total reflections	68221	1104474	467187
Unique reflections	125959	205057	61555
Wilson B-factor	29.2	25.9	22.3
Multiplicity	2.7 (2.0)	5.4 (1.8)	7.6 (7.2)
R_{merge} (%)	4.5 (71.5)	4.2 (63.4)	9.2 (75.4)
$CC_{1/2}$ (%)	99.7 (78.2)	100 (87.7)	99.7 (73.8)
CC* (%)	99.9 (91.6)	99.7 (97.2)	99.9 (92.2)
<I>/s(I)	9.3 (1.7)	100 (99.9)	11.1 (2.0)
**Refinement***			
Reflections: work/free	51981 (2744)/2673 (142)	110187 (3639)/5628 (186)	53240 (2793)/2729 (147)
R_{work}/R_{free} (%)	19.6 (24.2)/21.9 (27.5)	18.6 (22.8)/21.5 (24.3)	20.2 (25.2)/23.2 (30.4)
Number of TLS groups	3	6	4
Number of atoms			
Protein	3195	6407	3206
Ligand	11	186	15
Water	159	423	251
Average B-factors ($Å^2$)			
Protein	39.9	35.08	33.8
Ligand	53.6	56.69	46.1
Water	36.4	39.17	34.1
r.m.s.d.			
Bond lengths (Å)	0.007	0.006	0.007
Bond angles (°)	0.9	0.82	0.84
Molprobity†			
Favored	98.44% (384 aa)	98.2% (759 aa)	97.7% (381 aa)
Allowed	100% (390 aa)	100% (773 aa)	100% (390 aa)
Outliers	none	none	none
Clash score	99th percentile	99th percentile	99th percentile
Molprobity score	100th percentile	100th percentile	99th percentile
**RCSB ID**	9C6V	9C6W	9C6X

* Statistics calculated using PHENIX; highest resolution shells indicated in parentheses

† Calculated with the program MOLPROBITY

Table S2. Interactions between peptide NRVMLPKAA and HLA-B*39:06 residues

Complex	Residue	Atom	Polar contacts (atom; distance)	Van der Waals contacts	Water-mediated contacts
B*39:06/NRV	Asn1	N	Tyr7 (OH; 2.8 Å)	Tyr59, Asn63, Thr163, Trp167	
			Tyr171 (OH; 2.8 Å)		
		O	Asn63 (Nδ2; 3.1 Å)		
			Tyr159 (OH; 2.6 Å)		
	Arg2	N	Asn63 (Oδ1; 2.9 Å)	Tyr7, Tyr9, Val34, Cys67	
		Nε	Glu45 (Oε2; 2.7 Å)		
		Nη1	Tyr9 (OH; 3.0 Å),		
			Ser24 (Oγ; 2.7 Å)		
		Nη2	Glu45 (Oε1; 2.7 Å)		
	Val3	N	Tyr99 (OH; 3.0 Å)	Tyr9, Ile66, Asn70, Tyr99, Tyr159	
	Met4			Gln65, Ile66	
	Leu5	N	Asn70 (Oδ1; 3.0 Å)	Tyr9, Thr97, Tyr99	Asn70, Thr73
	Pro6			Phe116, Trp147, Val152	Asp74, Ser77, Trp95
	Lys7	Nζ	Glu76 (Oε2; 2.9 Å)	Thr 73	Lys146
		O	Lys146 (Nζ; 3.1 Å)		
			Trp147 (Nε1; 2.8 Å)		
	Ala8	N	Ser77 (Oγ; 2.9 Å)	Trp95, Thr143, Trp147	
		O	Asn80 (Nδ2; 2.8 Å)		
	Ala9	N	Thr143 (Oγ1; 2.9 Å)	Asn80, Lys146	

Table S3. Interactions between peptide NRVMLPKAA and HLA-B*39:01 residues

Complex	Residue	Atom	Polar contacts (atom; distance)	Van der Waals contacts	Water-mediated contacts
B*3901/NRV	Asn1	N	Tyr7 (OH; 2.8 Å)	Tyr59, Asn63, Ile66, Thr163, Trp167	
			Tyr171 (OH; 2.9 Å)		
		Oδ1	Arg62 (Nε; 2.9 Å)		
			Arg62 (Nη2; 2.9 Å)		
			Asn63 (Nδ2; 3.2 Å)		
		O	Tyr159 (OH; 2.7 Å)		
	Arg2	N	Asn63 (Oδ1; 2.9 Å)	Tyr7, Tyr9, Val34, Phe36, Asn63, Cys67	
		Nε	Glu45 (Oε2; 2.7 Å)		
		Nη1	Tyr9 (OH; 3.0 Å)		
			Ser24 (Oγ; 2.6 Å)		
		Nη2	Glu45 (Oε1; 2.7 Å)		
	Val3	N	Tyr99 (OH; 3.0 Å)	Tyr99, Tyr159	
		O	Asn70 (Nδ2; 3.1 Å)		
	Met4				
	Leu5	N	Asn70 (Oδ1; 3.0 Å)	Asn70, Thr73, Arg97, Tyr99, Asn114	Asn70, Thr73
	Pro6	O	Arg97 (Nη1; 3.0 Å)	Arg97, Trp147, Val152	
			Arg97 (Nη2; 2.9 Å)		
	Lys7	Nζ	Glu76 (Oε1; 2.5 Å)	Thr73, Glu76	
		O	Lys146 (Nζ; 3.0 Å)		
			Trp147 (Nε1; 2.8 Å)		
	Ala8	N	Ser77 (Oγ; 2.9 Å)	Ser77, Phe116, Lys146, Trp147	
		O	Asn80 (Nδ2; 2.9 Å)		
	Ala9	N	Thr143 (Oγ1; 2.8 Å)	Asn80, Cys84, Tyr123, Thr143, Lys146	Tyr123, Ala139, Thr143

Table S4. Positional scanning combinatorial library-based matrix describing 9-mer peptide binding to HLA-B*39:06

	Position								
Residue	1	2	3	4	5	6	7	8	9
A	0.496	2.85	0.625	1.25	2.75	0.468	0.794	1.19	306
C	0.784	0.091	0.770	0.792	2.84	0.813	0.319	0.256	7.42
D	0.722	0.125	0.205	0.695	0.548	0.476	0.128	0.468	0.417
E	2.93	0.311	0.161	1.98	0.124	0.942	0.269	1.49	0.429
F	6.35	0.113	6.34	1.67	2.18	30.5	0.938	2.78	0.563
G	0.455	0.387	1.09	1.45	0.526	0.497	0.142	1.50	19.2
H	6.85	2.93	2.50	0.897	0.464	0.485	0.410	22.1	2.01
I	2.77	0.123	1.41	2.40	3.16	0.424	0.555	1.091	7.01
K	0.891	0.108	1.62	1.20	1.71	0.270	0.126	0.898	0.469
L	0.901	0.091	1.73	1.45	2.53	0.862	0.649	1.66	1.73
M	3.09	0.575	3.38	0.975	40.9	0.948	0.840	0.803	0.392
N	4.13	0.262	1.74	1.60	0.720	0.510	0.517	1.46	0.492
P	0.654	0.614	2.31	1.40	0.643	0.426	12.5	8.19	1.71
Q	0.591	0.810	0.919	0.738	0.382	0.514	0.964	0.101	0.113
R	0.969	7.17	1.20	0.912	0.510	0.642	0.924	0.914	2.74
S	1.02	0.204	0.265	1.23	3.94	1.06	0.428	1.78	11.2
T	1.38	0.181	0.538	3.17	2.66	0.885	0.597	1.04	28.0
V	0.747	0.195	0.915	2.65	0.417	0.715	0.597	1.68	113
W	1.51	0.114	1.98	3.65	0.572	3.99	0.680	0.603	1.92
Y	2.21	0.127	0.863	2.64	0.418	0.635	0.696	0.217	0.456
Geomean	1.38	0.31	1.07	1.45	1.14	0.81	0.55	1.13	2.57
SD	1.9	1.7	1.4	0.85	8.9	6.7	2.7	4.9	70.7
SF	0.73	3.18	0.94	0.69	0.87	1.24	1.80	0.88	0.39
5-fold	2	11	2	0	2	1	4	3	8

Each positional scanning combinatorial library was tested for binding, and the data analyzed, as referenced in the text. Values shown represent the binding capacity of the corresponding library relative to average binding capacity of all pools. SD indicates the standard deviation between pools at the same position. SF is the specificity factor, representing the ratio of the average binding of the entire library to the average (geomean) of pools at the indicated position. The library average binding for HLA B*39:06 was 6974 nM. The number of pools at each position associated with a 5-fold increase or decrease in binding relative to the library average are also tallied. Dominant positions influencing binding capacity may be defined considering high SF, SD, and/or the number of 5-fold influences.

Table S5. Human insulin and G6Pase 2 peptides tested for binding to HLA-B*39:06, B*39:01, and B*38:01

Peptide source	Peptide	Position	Length
Ins	MALWMRLLP	1	9
	MALWMRLLPL	1	10
	LWMRLLPLLA	3	10
	WMRLLPLLA	4	9
	MRLLPLLA	5	8
	MRLLPLLAL	5	9
	MRLLPLLALL	5	10
	LLPLLALLA	7	9
	LALWGPDPA	14	9
	LALWGPDPAA	14	10
	ALWGPDPA	15	8
	ALWGPDPAA	15	9
	ALWGPDPAAA	15	10
	NQHLCGSHLV	27	10
	SHLVEALYL	33	9
	ERGFFYTPKT	45	10
	YTPKTRREA	50	9
	TPKTRREA	51	8
	RREAEDLQV	55	9
	GQVELGGGP	64	9
	QVELGGGPGA	65	10
	VELGGGPGA	66	9
	LGGGPGAGS	68	9
	GAGSLQPLA	73	9
G6Pase 2	HRNGVLII	5	8
	HRNGVLIIQ	5	9
	HRNGVLIIQH	5	10
	RNGVLIIQH	6	9
	IQHLQKDYRA	12	10
	QHLQKDYRA	13	9
	QKDYRAYYTF	16	10
	YRAYYTFL	19	8

Peptide source	Peptide	Position	Length
	YRAYYTFLN	19	9
	YRAYYTFLNF	19	10
	YTFLNFMSNV	23	10
	RNIFFIYFP	36	9
	IFFIYFPLC	38	9
	FQFNQTVGT	47	9
	TVGTKMIWVA	52	10
	VAVIGDWLN	60	9
	GHRPYWWV	77	8
	HRPYWWVQET	78	10
	RPYWWVQET	79	9
	QIYPNHSSP	88	9
	NHSSPCLEQF	92	10
	SPSGHAMGA	111	9
	SGHAMGASC	113	9
	HAMGASCV	115	8
	HAMGASCVW	115	9
	AMGASCVW	116	8
	AMGASCVWYV	116	10
	SHTVCGMDKF	132	10
	VCGMDKFSI	135	9
	CGMDKFSIT	136	9
	THFPHQVIL	173	9
	EAFEHTPGI	191	9
	FEHTPGIQTA	193	10
	EHTPGIQTA	194	9
	HTPGIQTA	195	8
	HTPGIQTAS	195	9
	QTASLGTYL	200	9
	TASLGTYL	201	8
	SLGTYLKTN	203	9
	TNLFLFLFA	210	9
	TNLFLFLFAV	210	10
	NLFLFLFA	211	8

Peptide source	Peptide	Position	Length
	NLFLFLFAV	211	9
	LFLFLFAV	212	8
	FLFLFAVG	213	8
	FLFAVGFYL	215	9
	FLFAVGFYLL	215	10
	VGFYLLLRV	219	9
	NIDLLWSVP	229	9
	IHIDTTPFA	249	9
	IHIDTTPFAG	249	10
	INSEMFLLS	273	9
	SCRGGNNYTL	281	10
	CRGGNNYT	282	8
	CRGGNNYTL	282	9
	CRGGNNYTLS	282	10
	FRLLCALTSL	292	10
	ALTSLTILQ	297	9
	SLTILQLYHF	300	10
	QLYHFLQI	305	8
	YHFLQIPT	307	8
	HFLQIPTHE	308	9
	THEEHLFYV	314	9
	THEEHLFYVL	314	10
	FYVLSFCKSA	320	10
	YVLSFCKSA	321	9
	CKSASIPLT	326	9
	SASIPLTVVA	328	10
	LTVVAFIPY	333	9
	VVAFIPYSV	335	9
	VHMLMKQSG	343	9

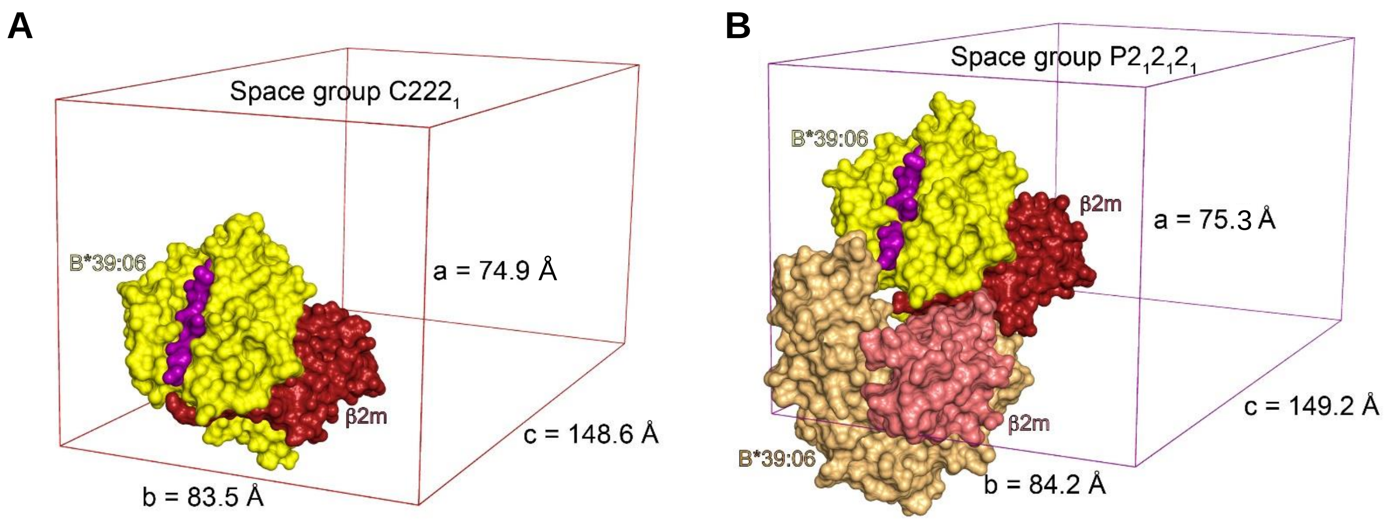
A
Space group C222$_1$
B*39:06
a = 74.9 Å
β2m
c = 148.6 Å
b = 83.5 Å
B
Space group P2$_1$2$_1$2$_1$
B*39:06
β2m
a = 75.3 Å
c = 149.2 Å
β2m
B*39:06
b = 84.2 Å

Figure S1

Figure S1. Structures of single-chain HLA-B*39:06/NRV obtained in two distinct space groups. *A*, Single-chain HLA-B*39:06/NRV (surface representation) with one molecule/asymmetric unit in space group $C222_1$. The HLA-B*39:06 heavy chain is shown in *yellow*, β2m in *red*, and the NRV peptide in *purple*. *B*, Single-chain HLA-B*39:06/NRV in space group $P2_12_12_1$ with two molecules/asymmetric unit. The HLA heavy chain and β2m are shown in two shades of *yellow* and *red*, respectively. The NRV peptide is shown in *purple*. Unit cell dimensions are indicated.

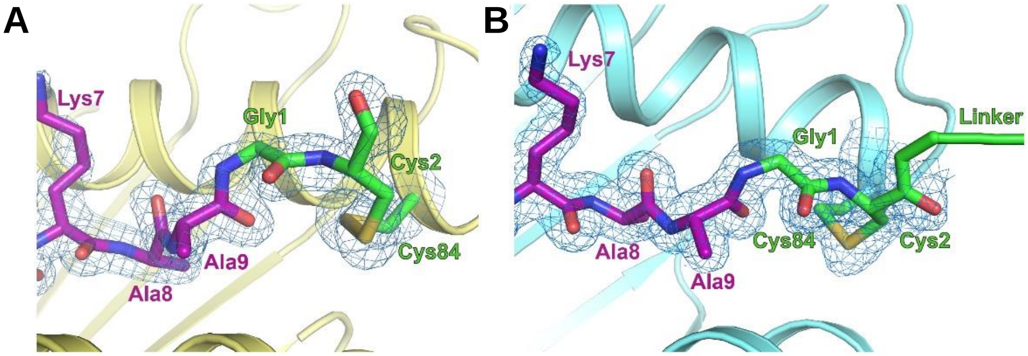
A
Lys7
Gly1
Cys2
Cys84
Ala9
Ala8
B
Lys7
Linker
Gly1
Ala8
Cys84
Cys2
Ala9

Figure S2

Figure S2. The engineered disulfide bond in the single-chain HLA-B structures. *A* and *B*, The engineered disulfide bond between Cys2 of the peptide-to-β2m linker and Y84C of the heavy chain of HLA-B*39:06 (*yellow*) (*A*) and B*39:01 (*cyan*) (*B*) is shown in *gold*. The NRV peptide (*purple*) and part of the linker sequence (*green*) are shown in stick representation. Omit maps contoured at 2.0 σ for the peptide and the linker are shown in *blue mesh*.

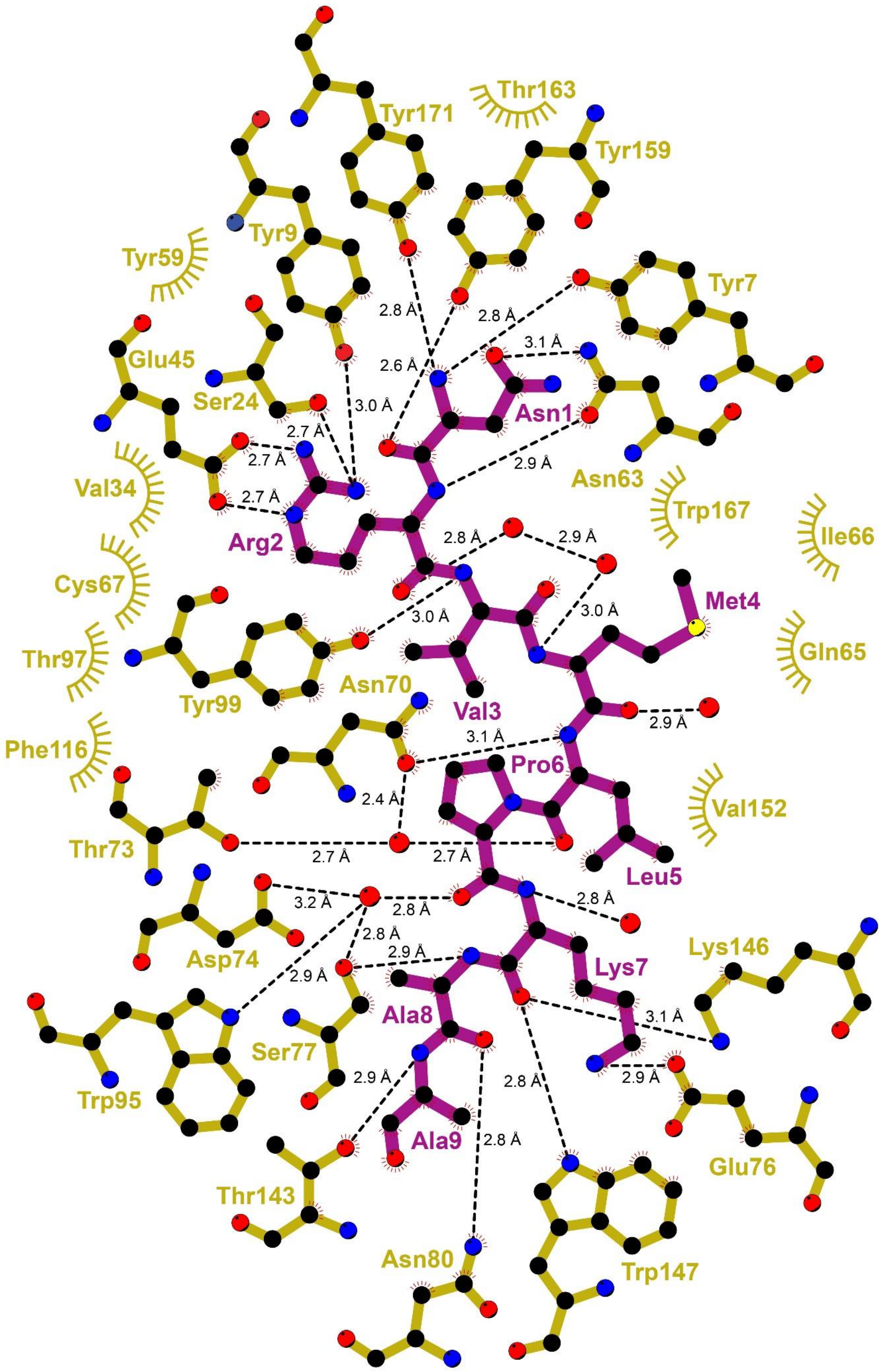

Thr163
Tyr171
Tyr159
Tyr9
Tyr59
Tyr7
Glu45
Ser24
Asn1
Asn63
Val34
Trp167
Ile66
Arg2
Cys67
Met4
Gln65
Thr97
Tyr99
Asn70
Val3
Phe116
Pro6
Val152
Thr73
Leu5
Asp74
Lys7
Lys146
Ala8
Ser77
Trp95
Ala9
Thr143
Glu76
Asn80
Trp147
2.8 Å
2.8 Å
3.1 Å
2.6 Å
3.0 Å
2.7 Å
2.7 Å
2.7 Å
2.9 Å
2.8 Å
2.9 Å
3.0 Å
3.0 Å
2.9 Å
3.1 Å
2.4 Å
2.7 Å
2.7 Å
3.2 Å
2.8 Å
2.8 Å
2.8 Å
2.9 Å
2.9 Å
3.1 Å
2.9 Å
2.9 Å
2.8 Å
2.8 Å

Figure S3

Figure S3. Interaction of HLA-B*39:06 with the NRV peptide. A detailed contact map displays interactions of the MHC heavy chain (*yellow*) with the peptide (*purple*). Selected water molecules are shown as red spheres. Potential hydrogen bonds are shown as dashed lines with distances indicated, and hydrophobic contacts are represented by an arc with spokes radiating toward the peptide. Small *red spokes* indicate corresponding atoms involved in intra- and intermolecular hydrophobic contacts.

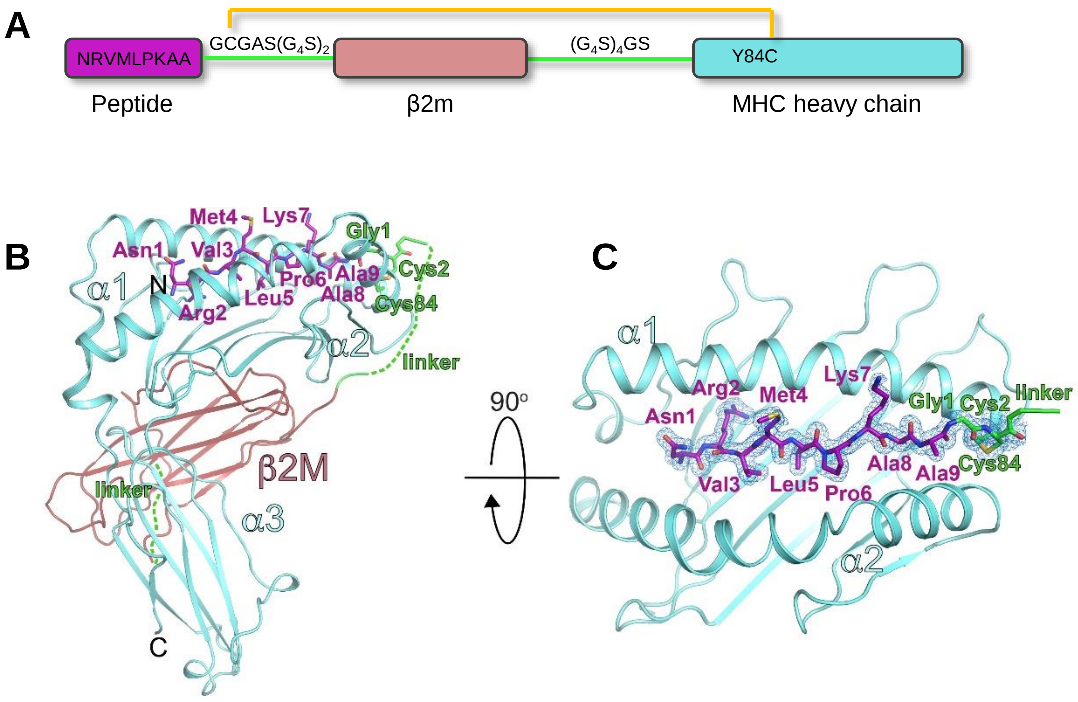
A
NRVMLPKAA
GCGAS(G4S)2
(G4S)4GS
Y84C
Peptide
β2m
MHC heavy chain
B
Met4
Lys7
Gly1
Asn1
Val3
Cys2
α1
N
Pro6
Ala9
Leu5
Ala8
Arg2
Cys84
α2
linker
β2M
linker
α3
C
90°
C
α1
Lys7
Arg2
Met4
Gly1
Cys2
linker
Asn1
Val3
Leu5
Ala8
Ala9
Cys84
Pro6
α2

Figure S4

Figure S4. Crystal structure of HLA-B*39:01/NRV. *A*, Schematic representation of the expression construct (not to scale). The peptide (NRVMLPKAA) is shown in *purple*, β2m in *red*, and the ectodomain of the HLA-B*39:01 heavy chain in *cyan*. Shown in *green* are linkers between the C-terminus of the peptide and the N-terminus of β2m and between the C-terminus of β2m and the N-terminus of the HLA-B*39:01 heavy chain. The engineered disulfide between the Cys of the first linker and Y84C of the heavy chain is depicted in *gold*. *B*, View of HLA-B*39:01/NRV showing the HLA-B*39:01 heavy chain (*cyan*), β2m (*red*), and the NRV peptide (*purple* and labeled; stick representation). Linkers are shown in *green* and are dashed if electron density was not visible. The engineered disulfide bond between Cys2 of the peptide-to-β2m linker and Y84C of the heavy chain is indicated in *gold*. The α1, α2, and α3 domains of the MHC heavy chain are labeled. N and C denote the location of N and C termini. *C*, An orthogonal view of the peptide-binding groove of HLA-B*39:01/NRV colored as in (*A*). Omit map corresponding to the NRV peptide is shown in *blue mesh* ($\sigma = 2.5$).

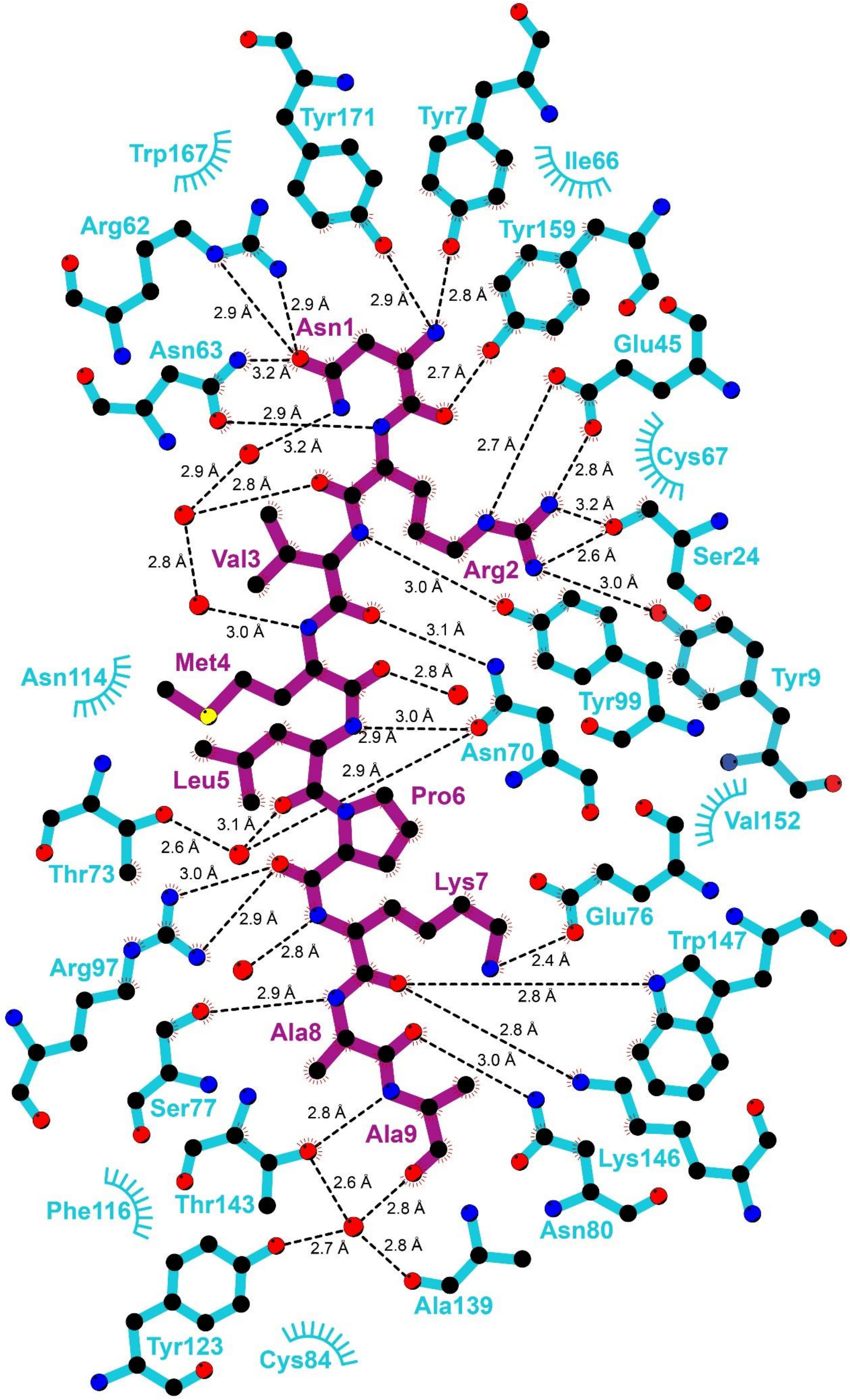

Tyr171
Tyr7
Trp167
Ile66
Arg62
Tyr159
2.9 Å
2.9 Å
2.9 Å
2.8 Å
Asn1
Asn63
3.2 Å
Glu45
2.7 Å
2.9 Å
3.2 Å
2.7 Å
Cys67
2.9 Å
2.8 Å
2.8 Å
3.2 Å
2.8 Å
Val3
Arg2
2.6 Å
Ser24
3.0 Å
3.0 Å
3.0 Å
3.1 Å
Asn114
Met4
2.8 Å
Tyr9
Tyr99
3.0 Å
2.9 Å
Asn70
Leu5
2.9 Å
Pro6
Val152
3.1 Å
2.6 Å
Thr73
3.0 Å
Lys7
Glu76
2.9 Å
Trp147
Arg97
2.8 Å
2.4 Å
2.9 Å
2.8 Å
Ala8
2.8 Å
3.0 Å
Ser77
2.8 Å
Ala9
Lys146
2.6 Å
Phe116
Thr143
2.8 Å
Asn80
2.7 Å
2.8 Å
Ala139
Tyr123
Cys84

Figure S5

Figure S5. Interaction of HLA-B*39:01 with the NRV peptide. A detailed contact map displays interactions of the MHC heavy chain (*cyan*) with the peptide (*purple*). Selected water molecules are shown as red spheres. Potential hydrogen bonds are shown as dashed lines with distances indicated, and hydrophobic contacts are represented by an arc with spokes radiating toward the peptide. Small *red spokes* indicate corresponding atoms involved in intra- and intermolecular hydrophobic contacts.
